# Feature Engineering for Surrogate Models of Consolidation Degree in Additive Manufacturing

**DOI:** 10.3390/ma14092239

**Published:** 2021-04-27

**Authors:** Mriganka Roy, Olga Wodo

**Affiliations:** 1 Mechanical and Aerospace Engineering Department, University at Buffalo, Buffalo, NY 14260, USA; mriganka@buffalo.edu; 2 Materials Design and Innovation Department, University at Buffalo, Buffalo, NY 14260, USA

**Keywords:** additive manufacturing, data-driven approach, fused filament fabrication

## Abstract

Surrogate models (SM) serve as a proxy to the physics- and experiment-based models to significantly lower the cost of prediction while providing high accuracy. Building an SM for additive manufacturing (AM) process suffers from high dimensionality of inputs when part geometry or tool-path is considered in addition to the high cost of generating data from either physics-based models or experiments. This paper engineers features for a surrogate model to predict the consolidation degree in the fused filament fabrication process. Our features are informed by the physics of the underlying thermal processes and capture the characteristics of the part’s geometry and the deposition process. Our model is learned from medium-size data generated using a physics-based thermal model coupled with the polymer healing theory to determine the consolidation degree. Our results demonstrate high accuracy (>90%) of consolidation degree prediction at a low computational cost (four orders of magnitude faster than the numerical model).

## 1. Introduction

**Figure 1:**
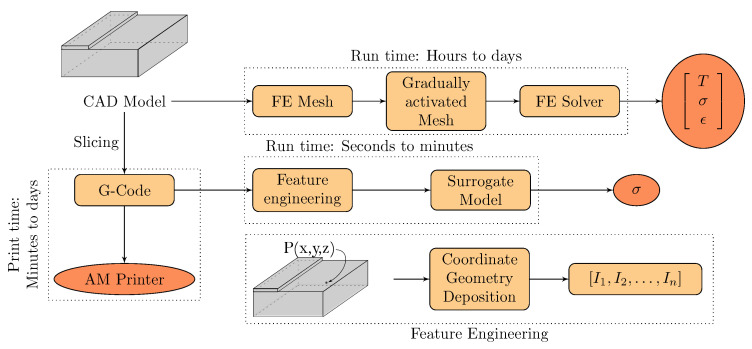
A schematic representation of numerical model, surrogate model, and printing process. In general, it takes a few minutes to hours to print a part depending on the size. Few exceptionally large parts require hours to print. However, it requires hours to days to simulate the same parts using traditional modeling. A surrogate model can predict the output in real-time.

High fidelity models (HFM) can capture the physical phenomena in many manufacturing processes with high accuracy. However, they also come with an inherent burden of high computational cost that impedes any practical optimization of the process or in-situ control. The finite element models [1,2,3,4,5,6] of the thermally driven processes in additive manufacturing belong to this category. Current state-of-the-art physics-based models require more time to simulate the underlying processes than to physically print and test the specimen (Figure 1). The major cost stems from the need to solve the large system of equations (i.e., transient heat equation) subjected to complex boundary conditions in AM. The mesh activation associated with the gradual deposition of small blocks or thin layers additionally makes the model numerically expensive. Finally, the computational burden is aggravated by the need to handle the associated boundary conditions that change in each time step. The cost of simulation grows exponentially with the size of the part geometry. Although various advanced numerical approaches [7,8] have been proposed to cope with the computational cost, these approaches still lack the speed required for real-time monitoring or optimization. For these reasons, surrogate models have been emerging as a computationally efficient alternative to the numerical models.

In recent years, there have been multiple efforts focusing on creating surrogate models to cope with the computational cost of the HFMs for AM [5,9,10,11,12,13,14]. In general, the data acquired from the high fidelity simulations or experiments are used for training the surrogate models (see Figure 1). A physics-based model focuses on capturing the underlying physics given a set of input parameters (e.g., all materials properties, system, and process variables). In the surrogate models, the focus is the opposite. Surrogate models aim to map the specific input set to the selected outputs, while significantly reducing the computational cost. Since the surrogate model is constructed with a specific objective, typically targeting the subset of input and output variables, a significant reduction in computational cost is possible. However, when the input or the output of SM is not a scalar but a high-dimensional vector, the SM may require significantly more data, more sophisticated statistical models, or might become impractical to build [15].

### Two Data Regimes and Complexity of the Surrogate Models in AM

In Table 1, we list the state-of-the-art surrogate models and compare and contrast the size of data, the inputs and outputs, and the model used. The SMs handle various processes in the AM family ranging from fused filament fabrication (FFF) to direct energy deposition (DED). We sort the models listed in Table 1 based on the size of data used for the training. The size can be as small as 60 data points when the output is a scalar (i.e., the pull-up stress in [9]) or as high as 250,000 for the time-series temperature prediction [12]. Three to four orders of magnitude difference in terms of size can be attributed to the high dimensionality of the output of SM. All models in the large data category handle high-dimensional vectors, either time-series data of temperature profile [16] or distortion field [13]. We follow the increasing data size order to discuss the various aspects of the models and motivate our approach. We grouped models into two categories: high- and low-volume data regimes.

The first category of models handles high-dimensional input and output and tends to employ deep learning methods. We consider the model by Mozaffar et al. [12] as the most complex model among these that we compare. The model has been built based on the data from the physics-based model of the direct energy deposition process (DED). The SM aims to predict the temperature at any point as a time series using a recurrent neural network (RNN). The input to the SM is relatively diverse and consists of the process parameters (laser power and scan speed), tool-path (distance from the deposition and relative time), and the geometry (the set of two shortest distances from the closest free surfaces). The RNN utilizes a large dataset (250,000 points) for training, and the reported training time was 40 h (100 epochs) on an Nvidia Quadro P5000 system. The model was tested on two separate datasets: (i) expanded time span; and (ii) dissimilar geometries. The performance of the model has been shown for selected points from several geometries. Moreover, the performance in terms of extrapolation into the expanded time (i.e., beyond training time) has been shown for a few randomly chosen points. The testing mean square error for a time span longer than the training time span was reported as $3.17\cdot 10^{-5}$. However, when tested on different geometry, the error was only reported qualitatively to be significantly higher. The reported reason behind the high error was attributed to the small number of features assigned to capture the geometry (two hand-picked parameters).

A broader set of input variables capturing the tool-path was proposed by Stathatos et al. [16]. Instead of using one distance from the deposition (as in [12]), this model aims to handle a more complex tool-path by engineering a set of variables that represent an intricate heat introduction pattern in a Laser-Based Additive Manufacturing (LBAM) process. The effect of the laser heating at any point is taken into account by dividing the neighboring area into a few concentric circles with increasing radius according to the Fibonacci sequence. The number of the heat source points within each circle acts as an input to the surrogate model. The proposed feature set captures the heat introduction associated with the tool-path in terms of density of deposition, and each circle designates the severity of the heat source. The model also addresses the effect of past trajectories by defining a heuristic cutoff time. The SM is based on artificial neural networks (ANN) and predicts the effect of the laser on the nearby region as the laser moves in space. The predictive power of the SM is showcased for multiple test trajectories. The model predicted the temperature and density with high accuracy (mean <1%) three orders of magnitude more quickly. However, the model was built and tested for trajectory on only one layer. Moreover, the model assumes an infinitely large system and does not consider the effect of cooling from free surfaces.

Another class of model was proposed by Francis et al. [13], who leveraged the convoluted neural networks to predict the distortion of the printed parts in LBAM processes. In that case, both the input and output of SM are high-dimensional vectors. The input consists of the thermal images obtained through the experimental measurements, and the distortion field is considered as the output of the model (measured experimentally through a 3D surface profiling system). The data for the model were collected in situ during the printing processes and consist of 21,818 thermal images (40 GB). The total time of data collection was 66 min. The reported time is much lower than the equivalent computational time of any other model. However, the authors reported a relatively long training time of 26 days (and 260 epochs) on a supercomputer. The paper recognizes the localized behavior of the printing process and its effect on distortion. In this area, a radius of importance was assumed to account for the localized behavior. The model demonstrated an accuracy within the tolerance (30 microns) of an AM machine in the trained region; however, higher error (56 microns) was reported in the extrapolation region. The thermal images acting as features, a large dataset, and a robust learning algorithm enabled the model to predict with high accuracy. However, the high computational power and time requirements for training, expensive sensor setup, and large data requirements pose an obstacle to the usage of the model. Moreover, capturing the thermal response at a high resolution during the fast printing process requires a complex sensor setup.

The second category of SMs lies in the low volume regime, which requires fewer data points to train. In this case, the SMs focus on relating the process parameters (e.g., laser intensity and print speed) to the part properties (e.g., melt pool depth and stress) [11]. In such cases, the choice of the input quantities is relatively straightforward. This is in contrast to the above-mentioned models handling geometry or deposition pattern and/or predict time-series output. Here, we compare two representative models [9,10]. For example, Tapia et al. [10] created a Gaussian process-based surrogate model. The goal of this SM was to predict the variation of the melt pool depth of the Laser Powder-Bed Fusion process (L-PBF) with respect to a few selected process parameters (e.g., scan speed, laser power, beam size). The selection of the input was unambiguous, as the quantitative measure of these process parameters is readily available. The uncertainty of the experimental data was captured through the statistical Gaussian process model. The model was created with process-oriented features and limited dataset, as shown in Table 1. The work reported the predicted melt pool depth for testing process parameters with high accuracy and speed.

Wang et al. built the surrogate model to predict the pull-up separation stress for printed parts in a stereo-lithography (SLA) process. The model was constructed from the data generated using finite element simulated pull-up stress distribution. The input to the model consists of a two-dimensional shape grid (15×15) of the cross-section. The grid captures the shape of each layer. The pull-up stress constitutes the output of the model. The model was trained using a relatively small dataset with 60 grids. The authors showcased their ANN-based model by predicting the pull-up stress at a fraction of time (compared to the physics-based model) with high accuracy.

In summary, in the area of AM, at the current state of the data-driven models, there is a lack of well defined and tested features. Several surrogate models have been proposed so far which either rely on the large volume of data to capture the correlation between high dimensional inputs and outputs or operate in a small data regime and focus on selected low dimensional inputs and outputs. This paper aims to close this gap and designs the features by gradually incorporating the characteristics of the physical processes occurring in manufacturing. This work aims to demonstrate that, given the medium size of the data available, one can construct the robust surrogate model by enriching the set of features with the characteristics of the underlying physical processes. We note that the underlying physical processes are well established with analytical solutions to the simplified cases available. This knowledge is leveraged to define a new set of features.

Our model builds on the ideas from our previous work [17], where we reported that a relatively simple SM (based on the shallow neural network) assisted with distance-based features. Our former model can predict the thermal behavior for relatively simple and similar geometries (differing in size rather than shape). We categorize our model in the medium data regime (12,000 data points—see Table 1). Our former model deals with the time series thermal profiles, an example of high-dimensional output. The model in this paper expands the previous model by handling more complex geometries but simpler output. We build our surrogate model to predict the degree of consolidation of the printed parts in the fused filament fabrication (FFF) process.

Our model utilizes physics-based features to improve both training and testing performance. To that end, we leverage the form of an analytical solution to the instantaneous heat source to engineer the set of features. Moreover, we capture the effect of the geometry by a set of weighted effective distances from the heat sources and the sinks within a localized neighborhood that we call heat influence zone (HIZ). In this way, we reduce the number of potential features influencing the thermal behavior and consolidation degree. Through systematic studies, we demonstrate the performance of our model that operates in the medium data regime and is generalizable between geometries of various complexities.

## 2. Problem Formulation

The focus of this paper is on the feature engineering for a robust surrogate model of the fused filament fabrication process predicting the consolidation degree at the interfaces between the roads and layers. In this paper, we build a surrogate model (SM) that is a statistical approximation of the computationally expensive numerical simulations. The numerical simulation encompass the heat transfer model coupled with the polymer healing theory to determine the consolidation degree between roads described in Section 3.1 and Section 3.2. Formally, a complex numerical simulation can be represented as a mapping function where the inputs (physical parameters) are mapped to the output (quantity of interest):

(1) y=f(x)

where *y* is the response of the numerical model (f) for the set of input parameters *x*. In this work, *y* is the consolidation degree and *f* is the thermal model coupled with polymer healing theory. In a general case, the inputs (x) are given as the materials properties, process and system variables (e.g., print speed, feed rate, deposition temperature, base heating, and ambient conditions), and deposition details (e.g., geometry, deposition pattern, and deposition size). We aim to build the SM (g) represented as:

(2) $$\bar{y}=g\left(\bar{x}\right)$$

where $\bar{y}$ is the statistical approximation of *y* and $\bar{x}$ is the engineered feature set. The statistical inverse problem of determining a computationally inexpensive *g* by reducing the difference between *y* and $\bar{y}$ is called training. The training is conducted with the data generated from the numerical model that serve as the “experience” of the model.

The key challenge is to engineer a set of features $\left(\bar{x}\right)$ that is robust enough to predict the consolidation degree, as the SM training is performed on limited data. We focus on building an SM that is capable of predicting the consolidation degree for complex geometries. In other words, we focus on capturing the effect of part geometries for nontrivial deposition processes. We aim to engineer a feature set $\left(\bar{x}\right)$ that encodes the geometry-related factors into the model *g*, ensuring high accuracy and speed.

## 3. Numerical Model and the Physical Background of the Manufacturing Process

In this section, the key physical characteristics of the layer-by-layer printing process are described followed by the physics-based numerical model used to generate data for the surrogate model. First, details of the physical phenomena that govern the thermal behavior are given. Next, the model of the consolidation degree and its link with the thermal behavior are discussed.

### 3.1. Heat Transfer Model

The physical phenomena that govern the thermal behavior can be partitioned in these categories: (i) heat introduction by deposited material; (ii) conduction within the deposited material; and (iii) convection and radiation from the evolving free surfaces. The thermal response of FFF during the printing process was extensively investigated by Costa et al. [3]. The heat conduction within the part is represented by a transient heat equation:

(3) $$\frac{\partial \left(\rho C_{p}T\right)}{\partial t}=\nabla \left(\lambda \nabla T\right)$$

where ρ is the density of the deposited material, *T* is the temperature, $C_{p}$ is the specific heat of the material, and λ is the thermal conductivity. The thermal processes in FFF are influenced by the convection and radiation from the evolving free surface of the printed parts. The free surface flux $\left(Q_{surf}\right)$, associated with the surface cooling, is given by

(4) $$Q_{surf}=h\left(T-T_{am}\right)+\kappa \left(T^{4}-T_{\infty }^{4}\right)$$

where *h* is the heat convection coefficient of the material measured at the ambient temperature $\left(T_{am}\right)$, which is assumed to be the constant printing chamber temperature during the printing process, and κ is the radiation coefficient. The reference temperature at an infinite distance $\left(T_{\infty }\right)$ is also assumed to be $T_{am}$.

To predict temperature profiles for the deposited layers, Equation (3) is solved numerically (finite element method) with an initial thermal condition of deposition temperature and boundary conditions for surface flux (Equation (4)). The free surface is redefined at each time step with the boundary conditions applied. More details on the model are included in our other work [18]. The model was experimentally validated in our previous study [6]. The data are used for the training and testing of the surrogate model.

We design the features (see Section 4) by leveraging the analytical solution of the heat equation (Equation (3)) for the problem of instantaneous point source (Q) on an infinite medium [19]. The analytical solution is given by Equation (5):

(5) $$\Delta T\left(d,t\right)=\frac{Q}{{\left(4\pi \alpha t\right)}^{3/2}}exp\left(-\frac{d^{2}}{4\alpha t}\right)$$

where α is the thermal diffusivity. In AM processes, the point sources can be superimposed to model the cumulative effect of the heat. However, the complex boundary conditions associated with the cooling surfaces limit the treatment of the part as a semi-infinite medium. Nevertheless, we propose to leverage the analytical solution to design the feature related to the heat input and loss. Although the relative effect of heat sources and sinks on the temperature and consolidation degree may differ, the contributions depend on the distances. The intuition behind the feature engineering is explained in greater detail in Section 4.

### 3.2. Thermoplastic Consolidation

The second element of the numerical model involves consolidation degree calculations. The bonding strength is computed at the interfaces between the roads constituting the printed part. The consolidation occurs at the contact surfaces of the roads, also known as deposition tool-paths. The polymer chains diffuse across the heated interface. This process occurs at a temperature higher than the glass temperature $\left(T_{g}\right)$. This phenomenon is also known as thermal fusion bonding [20]. The time required to achieve the ultimate fusion (or bond strength) strongly depends on the temperature profile. If the interface between two roads is kept at an elevated temperature for an adequate time, the maximum fusion can be achieved. In a less ideal condition, partial bonding is achieved and may lead to fracture [21,22]. Hence, to determine the bonding strength, the temperature profile needs to be established. Once the temperature profile is available, the bond strength is computed using the polymer healing theory.

The bonding theory for the non-isothermal condition [23] is formulated as:

(6a) $$\gamma {\left(t\right)}^{1/2}={\left[\sum_{i=1}^{n}\left(\frac{t_{i+1}^{1/2}-t_{i}^{1/2}}{{\tau_{r}^{*}}^{1/2}}\right)\right]}^{1/2}$$

(6b) $$\frac{\sigma (t)}{\sigma_{\infty }}=\left\{\begin{array}{c} \gamma {\left(t\right)}^{1/2}\;\gamma {\left(t\right)}^{1/2}<=1\\ 1\;\;\gamma {\left(t\right)}^{1/2}>1 \end{array}\right.$$

where γ(t) is the ratio of the diffused length with respect to the length of the polymer chain at time *t*, σ(t) is the achieved bond strength at time *t*, $\sigma_{\infty }$ is the bulk strength or the ultimate bond strength, and *n* is the number of time intervals used to compute the bonding. For each time interval $\Delta t=t_{i+1}-t_{i}=t/n$, the reptation time $\tau_{r}^{*}$ is computed for the average temperature $\left(T_{i}^{*}\right)$ of the interval by the Williams–Landel–Ferry (WLF) equation [24].

(7) $$\tau_{r}\left(T\right)=\tau_{r}\left(T_{ref}\right)\times \exp\left(\frac{-C_{1}\left(T-T_{ref}\right)}{C_{2}+\left(T-T_{ref}\right)}\right)$$

where $C_{1},C_{2}$ are the WLF shift coefficients and $T_{ref}$ is the reference temperature. The coefficients are material dependent. The shift coefficients for ABS plastic were experimentally determined by Bartolai [25] using parallel shear rheometry.

## 4. Feature Engineering for the SM of Consolidation Degree

For any data-driven model, the choice of features is a critical element. Designing the features constitutes a major part of the modeling process. For complex systems with unknown or partially understood underlying processes, many features are chosen, followed by feature selection processes (wrappers and filters). There has been considerable work in the field of machine learning regarding feature selection [26,27] and engineering [28,29,30]. The primary aim of feature engineering is to reduce the computational cost, increase the accuracy and generalizability, and avoid over-fitting.

*Features in the AM Surrogate Models*

The features are proposed for the exemplar SM models in AM, which is fused filament fabrication. To capture the effect of the geometry or deposition path, different approaches have been explored including 12×12 grid-connection of a 2D layer [9], 2D images [11,13], the shortest distances from the free surfaces [12], trajectory descriptors [16], and process parameters [13]. The choice of features related to the geometry is dependent on the AM process under consideration. For a road-wise deposition process, such as thermally driven FFF, for each point in the part, Mozaffar [12] chose two distances to the nearest free surfaces. The relatively small feature set captures the effect of part geometry in terms of the cooling from the free surfaces. Such a choice of features has only been shown to perform well for simple geometries. Another approach was taken for the stereolithography apparatus (SLA) process in [9], where deposition is layer-wise. For each layer, the laser is used to draw a shape on to the surface of the already formed layers. The geometry of each layer was represented as a two-dimensional grid with 15×15 discrete points. The grid encoding the connectivity matrix was used as an input to SM that aimed to predict a scalar value of pull-up stress for a given layer. The relatively small size of the input (15×15) and output (1) led to a good performance of the training and testing using small data.

The feature capturing the effect of the printing pattern is even less explored. Only one work was found to propose a parameterization of intricate printing patterns. Stathatos et al. [16] introduced a trajectory descriptor for the LBAM process. The descriptors capture the effect of the heat sources that accounts for the relative distance and the relative time of the heated depositions. The authors demonstrated a highly accurate performance of the surrogate model for complex 2D scanning patterns. However, the model was trained and tested using 2D scanning patterns on a semi-infinite medium and only one layer. The expansion of the feature definition into 3D processes and defining input for the finite print boundaries are still to be advanced.

### 4.1. Limit the Number of Defined Features by Introducing a Heat Influence Zone

The major observation that drives the feature engineering in this work is related to the local characteristics of the deposition process. We observe that there exists a finite zone where the effect of the manufacturing-related processes is significant. We call this zone a heat influence zone (HIZ) [17] and define it as the maximum extent within which a heated deposition causes a significant thermal change within the printed part. We limit the distances related to the features to the extent of the HIZ. For the HIZ to be useful, it should be independent of the geometry and dependent only on the material properties and the deposition parameters. In our other work [17], we showed a conservative estimation of the HIZ. Here, the transient heat equation inside a semi-infinite part is used. The extent of the HIZ for ABS thermoplastic has been estimated to be 0.8 mm (4 layers). This estimation corresponds to the zone within which the temperature increase from a deposition is at least ΔT=10.5°C. Intuitively, beyond this zone, the thermal effect of the heat source and heat sinks should be negligible.

### 4.2. Two Major Pieces of Information: The Distance and the Relative Time

The major factors affecting heat dissipation and heat introduction in a system are the distance of the heat sinks and sources as well as the relative time of the heat source introduction (i.e., time of deposition). Hence, we choose these two factors to capture the effect of the geometry and the deposition pattern on the consolidation degree. Consequently, all features are derived from the distances from/to heat sources and sinks and the relative times of the deposition.

### 4.3. Designed Features

We design the features based on the underlying physics of the heat transfer by focusing on the heat sources and heat sinks that depend on the geometry, and the deposition pattern (see Figure 1). Heat sinks are associated with the part geometry and location of the cooling surfaces, while heat sources are associated with the printing pattern. In Table 2, we list the final set of features used in our model. In total, we derive five features related to the heat sources: (i) effective area; (ii) weighted effective distance; (iii) relative time of layer deposition; (iv) relative time of subsequent road deposition; and (v) relative time of previous road deposition. Additionally, we define two features related to heat sinks: one feature related to the free surfaces and one capturing the effect of the base. To calculate each feature, we consider only the distances and relative time related to the depositions within HIZ. Below, we discuss the features by categorizing them into geometry and printing pattern related features, respectively.

#### 4.3.1. Geometry-Related Features

Two sets of features related to the geometry are considered here: features related to the heat sources and features related to the heat sinks. The heat sources are represented by three factors: (i) the normalized area of deposition within the HIZ, where the area is normalized by the area of the HIZ; (ii) the weighted effective distance; and (iii) relative time of heat introduction. The heat sinks are represented by factors related to their distance from the point. Three categories are considered: the side free surface, top free surface, and base surface. The side and top free surfaces always act as a heat sink. However, the heated base may act as a heat sink or a source, depending on the part temperature.

**Figure 2:**
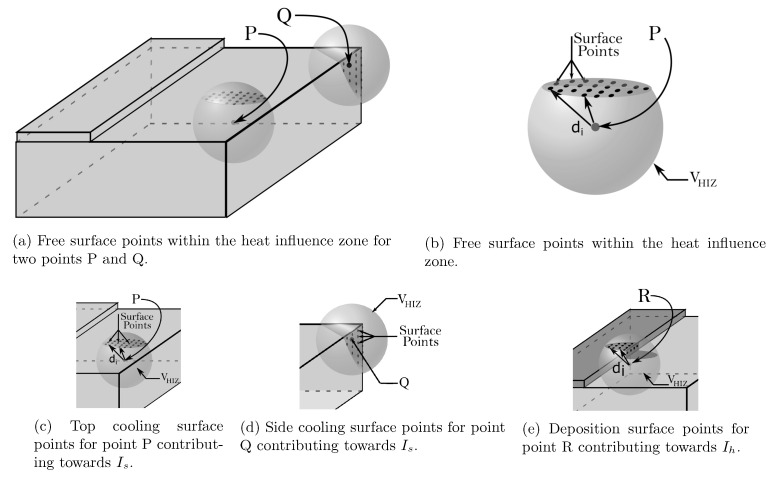
Surface points constituting the heat sinks for internal points P Q, and R. The spheres with radius $L_{HIZ}$ denote the HIZ around the points. The surface points are denoted by black circles.

We begin by explaining the feature related to the distances from the free surfaces. To capture the collective effect of the cooling from the free surfaces, we integrate over the local free surfaces within the HIZ. Figure 2 depicts the visual interpretation of this feature. For any point (P) of interest within the printed part, the influence from any free surface (top or side) is represented as:

(8) $$I_{s}=\sum_{i=1}^{N_{surf}}exp\left(\frac{-d_{i}^{2}}{4\alpha }\right)$$

where $N_{surf}$ is the number of uniformly distributed points on the surface that are within the HIZ and $d_{i}$ is the shortest distance from the point (without crossing voids). The number of discrete points used to compute the feature is chosen to match the discretization of the numerical model. The exact form of the feature is inspired by the closed-form solution of the transient heat equation with an external point source on a semi-infinite body [19]. In this case, rather than simply computing the shortest distances, we additionally normalize the distance with the heat diffusion length and weight the distance through the exponential function. In this way, the free surfaces located at a short distance from a given point are considered more influential than free surfaces at a longer distance. In the initial tests (see Results Section), we showcase that defining features using the shortest distance without the weighting function does not provide a good performance of SM. We report a superior performance of the SM when the weighting function is included in the feature.

An analogous form of the feature is leveraged to capture the effect of the base:

(9) $$I_{b}=exp\left(\frac{-d_{b}^{2}}{4\alpha }\right)$$

where $d_{b}$ is the shortest distance of the point from the base. However, this time only one distance is computed and weighted in an analogous way as in the previous feature.

The normalization and weighting of the distances is the major innovation of this work in the area of features. In Section 5, we analyze how the choice of the features affects the training convergence of the SM.

#### 4.3.2. Printing Pattern Related Features

**Figure 3:**
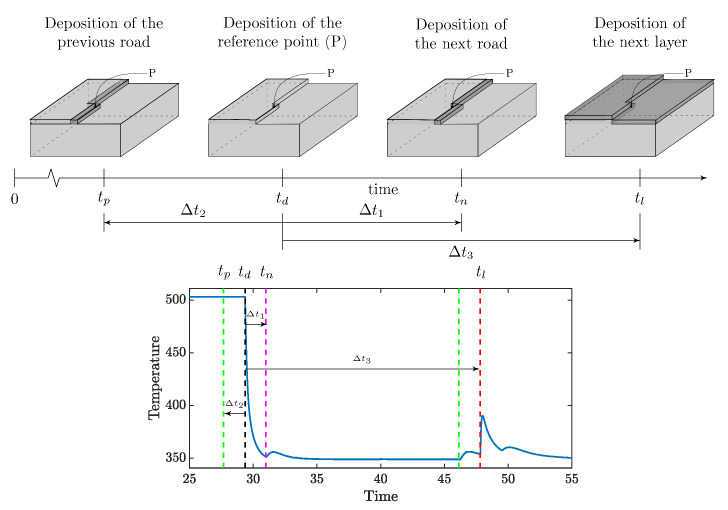
Feature representation of the deposition pattern. For a point P on the interface of a deposition, the three features representing the deposition pattern is denoted by the three time spans $(\Delta t_{1},\Delta t_{2},$ and $\Delta t_{3})$. The darker deposition represents the deposition occurred in the time span. The bottom panel depicts the temperature profile for a point *P* with features marked on the profile. The curve has been obtained through a computational model of heat transfer for the deposition process. Note that the features correspond to the characteristic points on the temperature profile.

The features related to the printing pattern directly affect the temporal evolution of the heat source. We use the following features to capture the dynamics of the pattern:

- Next road influence $\left(I_{n}\right)$ depends on the time at which the adjacent next road is deposited.Consider a point P on a given road, as shown in Figure 3, and its adjacent deposition. The relative time of deposition of the next road affects the heat transfer. Intuitively, if the relative time is long, the point gets sufficient time to cool down, and the heat retention will be lower. Thus, the temperature difference between the next road and *P* would be higher, and, in terms, heat flow would be higher, which causes high peaks. The effect of the next road can be quantified as:
  (10) $$I_{n}=\frac{1}{|t_{d}-t_{n}|}=\frac{1}{\Delta t_{1}}$$
  where $t_{d}$ is the time of deposition of point P and $t_{n}$ is that of the next road.
- Previous road influence $\left(I_{p}\right)$ depends on the time at which adjacent previous road was deposited. The previous roads also contribute to heat flow out of P. If the relative time of the previous road deposition $\left(t_{d}-t_{p}\right)$ is high, it will be at a low temperature, and higher heat will flow from *P* to the road compared to a more recently deposited one.
  (11) $$I_{p}=\frac{1}{|t_{d}-t_{p}|}=\frac{1}{\Delta t_{2}}$$
  where $t_{p}$ is the time of deposition of the neighboring previous deposition.
- Layer influence $\left(I_{l}\right)$ is the feature capturing the effect of the relative time of deposition of the next layer. This is one of the most influencing parameter as the most significant contributor to reheating is the subsequent layer depositions. The relative time dictates the heat accumulation. Considering the time of deposition of the next layer to be $t_{l}$, the influence is given as:
  (12) $$I_{l}=\frac{1}{|t_{d}-t_{l}|}=\frac{1}{\Delta t_{3}}$$

Note that all time differences are given with respect to the time of deposition for the point of interest (here *P*).

## 5. Results

In this section, the data generation is described first. Then, the analysis of the model robustness and their link to the increasing complexity of the features is provided. Our analysis supported by the results demonstrates the robustness of our approach for nontrivial geometries in terms of part geometry, printing pattern, and material behavior.

### 5.1. Data Generation

**Figure 4:**
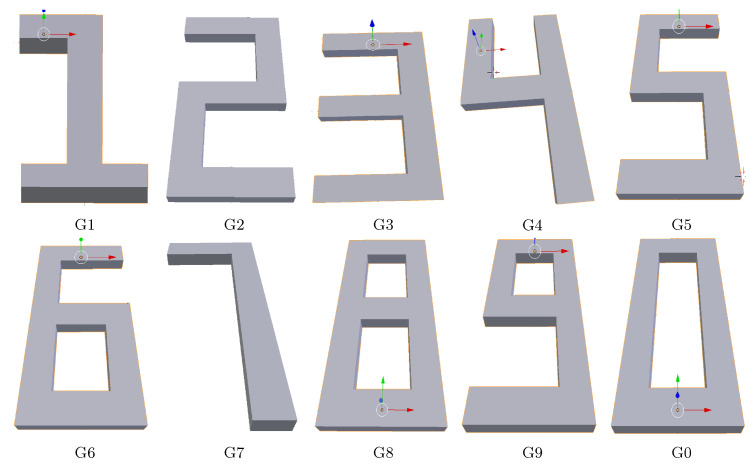
CAD models of the complex simulations. Each geometry is eight layers thick and has distinct printing pattern.

**Figure 5:**
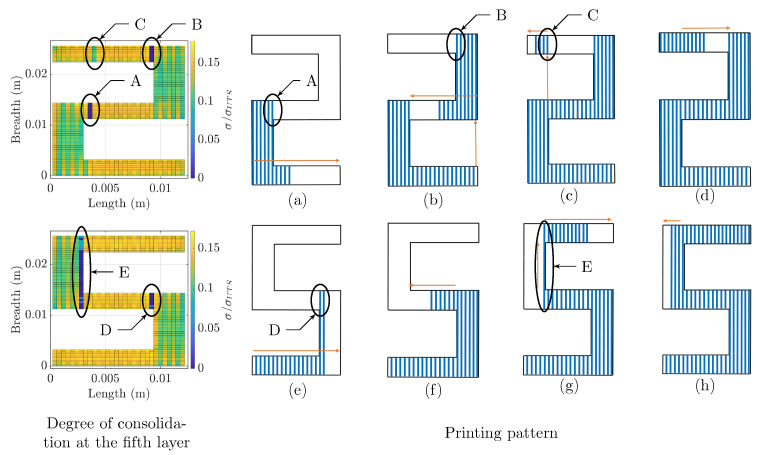
Effect of printing pattern over consolidation degree in similar geometry. **Note**: The two geometries are the same, where the difference lies only in the printing pattern. The first column depicts the distribution of the consolidation degree while the rest of the figure demonstrates the printing pattern. Panels (**a**–**h**) depict the details of printing pattern used. The blue lines represent the individual roads and the orange arrows direct the deposition progress (view in color). The difference in the printing pattern creates the consolidation artifacts marked by Regions A–E.

**Figure A1:**
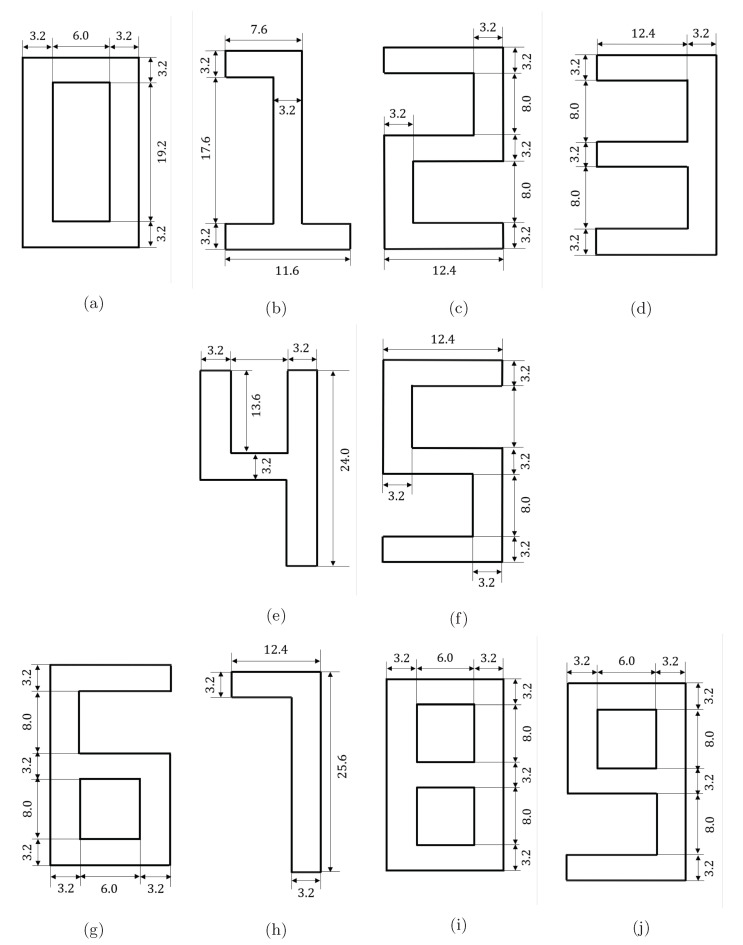
Schematic diagram of the digits (not to scale), panels **a**–**j** depict digits 0–9. All the dimensions are in mm scale.

The numerical model (Section 3) was used to simulate the layer-by-layer deposition process for a series of geometries with a nontrivial printing pattern. The ten Hindu–Arabic numerals (0–9) were chosen as part geometries, as they have sharp corners while having dissimilar geometries. For each geometry, a Gcode was generated using Cura^®^. CAD representations of all geometries, generated using Blender^®^, are shown in Figure 4, and an example printing pattern for digit five and two is depicted in Figure 5. Each digit consists of eight layers (each 0.2 mm thick) with the cross-section area on an average of 1.2 cm along the x-axis and 2.4 cm along the y-axis (the dimensions differ slightly between digits-more details are given in Appendix A, Figure A1).

Given Gcode, we generated the FEM computational mesh and the activation protocol simulating the quasi-continuous discrete deposition. For each discrete point on inter-layer and inter-road surfaces (between depositions), the thermal history was extracted from the FEM simulation. Next, the reptation theory was applied to the thermal history to compute the degree of consolidation, as explained in Section 3.2. The bonding degree, expressed as the ratio of the bond strength to the ultimate strength, is considered as the output of the surrogate model. The schematic is depicted in Figure 1.

In total, 87,808 data points were generated for the ten geometries. Five different geometries (one, two, four, six, and eight) were used for the training of the SM, which constitutes 42,912 data points. The size of data used in training places our model in the medium data size category.

### 5.2. Nontrivial Behavior for Relatively Simple Geometries

Before explaining the training process, we explain the nontrivial material behavior associated with our part geometries and printing patterns. An example distribution of the consolidation degree is depicted in Figure 5. We use two digits (two and five) to demonstrate the nontrivial behavior of the deposition pattern and consolidation for the seemingly similar geometries. Although the geometry of these two digits is very similar (rotational symmetry), the printing pattern is significantly different. This has implications on the distribution of the bonding degree.

Figure 5 depicts the distribution of the consolidation degree for a representative layer (Layer 5) along with the deposition pattern. For both digits, the printing process starts from the left bottom corner and proceeds right. Blue lines mark the sequence of the road depositions, while orange arrows indicate the changes in the printing pattern. The bonding degree is neither uniform nor continuous over the geometry. This occurs due to the nontrivial printing pattern that induces discontinuities in deposition that result in the discontinuous distribution of the consolidation.

In the left panel of Figure 5, five zones with a low consolidation degree are marked as A–E. The location of poorly consolidated zones in digit two (Zones A–C) is not consistent with that of digit five (D–E). The difference in the distribution of the low consolidation regions is a direct consequence of the difference in the deposition pattern. For example, in digit two, Segment A (as shown in the figure) has a low consolidation due to a delayed deposition of the adjacent subsequent road (next road). Specifically, the poor consolidation is attributed to tool-path, specifically the long time lag between the adjacent depositions and the associated heat transfer, resulting in the lower temperatures and reduced consolidation. As depicted in Figure 5a–c, after the deposition of material in Section A, the liquefier moves to the bottom and right part of digit two. The changes in the deposition are marked in the panels of Figure 5 with orange arrows to facilitate tracing of the changes in the deposition. A similar situation occurs for Segments B–E. A discontinuity in the printing pattern (time lag in deposition) translates to the discontinuity in the consolidation degree. Even though the low consolidation zones do not occur at the same position of the two similar geometries, the reason behind the phenomena is the same.

On the other spectrum of the consolidation degree, the higher consolidation regions are found in the areas with narrower breadth than the broader ones. Even though points in the narrower regions are potentially affected more by the cooling surfaces, the rate of heat introduction due to the next road deposition is relatively higher than the heat loss. The positive balance results in higher heat retention, and, consequently, higher consolidation. In the regions of broader breadth, we observe an alternating high and low consolidation due to an alternating print direction.

Moreover, the low consolidation regions occur due to the low thermal energy at temperature below $T_{g}$. Only above that temperature is the consolidation initiated. These characteristics pose challenges for the regressive models. To address this challenge, we build the surrogate model in two steps. In the first step, we first classify the data into two categories: unconsolidated points (σ(t)=0) and partially consolidated points (σ(t)>0). In the second step, the regressor model is used to predict the degree of consolidation only for the points with partial consolidation.

### 5.3. Technical Details

The data from the numerical model were generated on a 20 CPU system (Intel^®^ Xeon^®^ E5645 @2.40 GHz with 48 GB RAM). For the classification, decision trees were used to classify points that are fully consolidated and unconsolidated. Decision trees demonstrated the highest accuracy among other classifiers that were tested (e.g., SVM, LDA, QDA, and Gaussian SVM). The training was done on a four-core processor (Intel^®^ core i7-4790 CPU @ 3.60 GHz) with 12 GB RAM in parallel for 95 s. For partially consolidated points, the regression model was constructed. Here, a single-layered shallow neural network was constructed due to its low training time and high speed while reporting a high accuracy of prediction. The final designed neural network was trained on a 20-core processor (Intel^®^ Xeon^®^ E5645 @2.40 GHz with 48 GB RAM) and required only 10 s. Our training time is significantly lower than other models (see Introduction).

### 5.4. The Architecture of Our Surrogate Model

**Figure 6:**
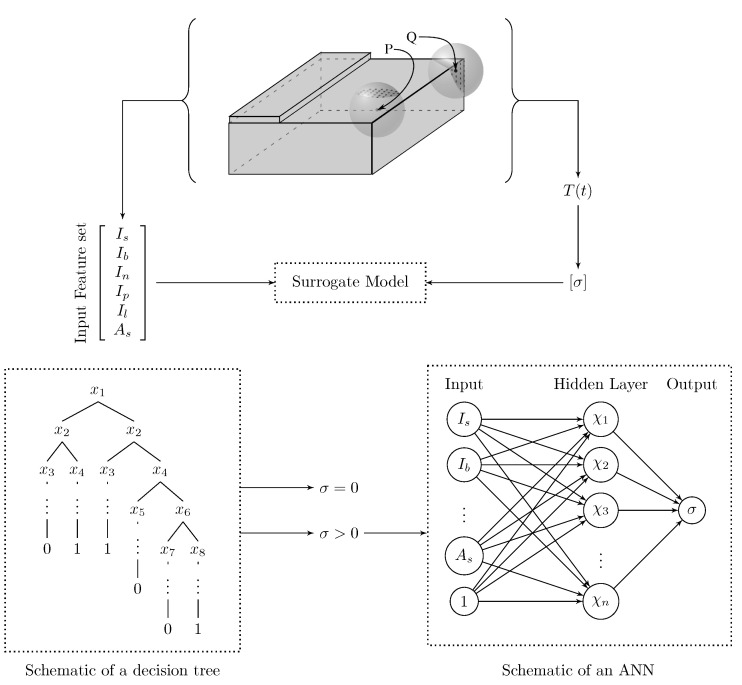
Structure of the surrogate model. The surrogate model consists of a decision tree followed by a single layer shallow artificial neural network.

The architecture of the SM is based on the problem definition and performance. The design of the model is depicted in Figure 6. Our model consists of two steps. In the first step, we build the decision tree-based classifier to distinguish the consolidated and unconsolidated points, as shown in the figure. The $x_{i}$ are the branch condition and 0 and 1 are the output corresponding to unconsolidated points and consolidated points. In the second step, we train the artificial neural network (ANN) regression model predicting the degree of consolidation of the consolidated points. As depicted in the figure, the ANN is trained with the same input set as the regression tree. The figure depicts the schematics of the ANN, where $\chi_{i}$ is the weighted sum of the input set and σ is the output (consolidation degree). The SM is trained and tested following the pipeline described below. First, the output is generated through a numerical model described in Section 3. The input to our SM is generated by using the Gcode, as described in Section 4. The repeated entries are removed from the training dataset. To train the decision tree (Step 1), we use both consolidated and unconsolidated data points since the aim is to differentiate between consolidated and unconsolidated points. However, to train the neural network (Step 2), only the data corresponding to the consolidated points are used.

The neural network was chosen with the following parameters:

- Type: Single layer shallow neural network (Figure 6).
- Regularizer: Bayesian regularization
- Loss function: Bayesian loss function [31]
- Optimizer: Levenberg–Marquardt
- Number of hidden nodes: 25
- Target training NMSE: $10^{-5}$

The parameters are chosen in an iterative fashion to accommodate faster convergence in training while avoiding over-fitting.

### 5.5. Testing

**Figure 7:**
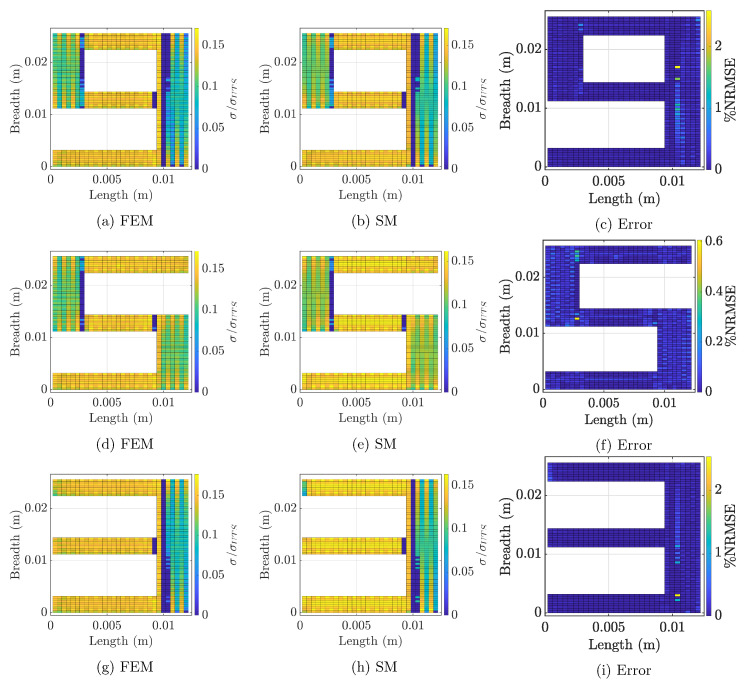
Comparison of the predictive power of the SM for complex geometry: (left) the distribution of the consolidation degree predicted using physics based simulation; and (right) the consolidation degree predicted by the SM.

We trained a series of surrogate models for different configuration of features (see next subsection) and then utilized each model to predict the consolidation degree for each layer of the tested geometries. Figure 7 reports the consolidation degree of an internal layer (Layer 5) predicted through the physics-based numerical model (the first column), surrogate modeling (the second column), and the associated normalized error (the third column) for testing geometries nine, five, and three. The results of SM correspond to the final selection of the features (see Table 2).

Figure 7 depicts the distribution of the consolidation degree with the nontrivial pattern across the layer of the print. The distribution is consistent between the numerical model and our surrogate-based predictions. The surrogate model predicts the consolidation degree for test geometries with high accuracy, as shown in the figure. Error distribution over the cross-section is random with reported error below 5%. In the studied geometries, several sections exist with unconsolidated points. For example, in Figure 5, a few such sections are marked. This is due to excessive cooling and non-optimal deposition pattern. For the selected geometries, the printing pattern plays a major role in the consolidation degree. Even though some sections of the parts achieve higher consolidation, it is still far from the ultimate strength (maximum 20% of the $\sigma_{UTS}$ is achieved). Such behavior is associated with the large size of the part. Nevertheless, the data exhibit a wide range of thermal behavior and constitute a good testing set for the feature engineering analysis.

Our surrogate model is built to augment the speed of the numerical model while maintaining a certain level of accuracy. While the accuracy of the surrogate model is visualized qualitatively in Figure 7, a quantitative evaluation is presented in Table 3. The capabilities of the surrogate model are evaluated in terms of both speed and accuracy for the numerical model for both testing and training geometries.

The model was tested with the data from the digits three, five, seven, nine, and zero. Each row of the table provides the run-time for both the numerical model and the SM, and it provides the accuracy of the SM. The models were executed in parallel in a 20 CPU system, and the prediction time was recorded for each geometry. The time of computation for the numerical model consists of the cumulative time from the thermal model and the reptation theory, whereas the surrogate model can predict the consolidation degree directly from the input parameters. We observe the higher average accuracy (Equation (13)) in the training geometries than the testing geometries, which is typical for machine learning models.

(13a) $$\sigma_{error}=\left\{\begin{array}{c} \frac{\left(\sigma_{p}-\sigma_{a}\right)}{\sigma_{a}}\;\sigma_{a}>0\\ 1\;\;\sigma_{a}=0\;and\;\sigma_{p}\neq \sigma_{a}\\ 0\;\;\sigma_{a}=0\;and\;\sigma_{p}=\sigma_{a} \end{array}\right.$$

(13b) $$error=\frac{1}{N}\sqrt{\sum_{i=1}^{N}{\left(\sigma_{err}\left(i\right)\right)}^{2}}$$

(13c) accuracy=(1−error)×100

Equation (13) provides the details of the error calculation. Here, $\sigma_{p}$ is the bonding degree predicted by the model and $\sigma_{a}$ is the bonding degree derived from the physics based analysis.

Among testing geometries, the highest accuracy is recorded for digit five (95%), and the lowest accuracy is recorded for digit seven (89%). The surrogate model required only few seconds to compute the consolidation degree for the entire part (see Table 3). Collectively, our surrogate model is four orders of magnitude faster than the numerical model while maintaining about 90% accuracy. It is noted that the execution time of the numerical model is exponentially increasing, but the SM is linear in nature. The statistics summarized in Table 3 showcase the computational superiority of the surrogate model.

### 5.6. Feature Engineering Analysis

The results presented in the previous subsection were predicted using the final set of features. In this subsection, we analyze the accuracy and complexity of the SM for several configurations of the features. This analysis and the gradual change in the features lead to the final model with high accuracy.

We perform a series of seven experiments, which we marked as E0–E6. In each experiment, we increase the complexity of the features (e.g., by adding the weighting function of the feature) or change the complexity of the data used in the model and quantify the accuracy of the model for the testing geometries (G3, G5, and G9). The details of the gradual evolution of the features is presented in Columns 2–4 of Table 4. The representation of the features corresponding to the distance from the cooling surfaces and the base is given in the second column. The size of the HIZ is varied to verify the initial assumption of its extent (0.8 mm), and it is given in the third column. Additional features are added in later experiments to represent the deposition pattern, which is given in the fourth column. The complexity of the dataset in terms of the boundary points and the unconsolidated (σ=0) points is given in the fifth and the sixth columns, respectively. The complexity of the shallow ANN is measured in terms of the number of hidden nodes and is given in the seventh column. The corresponding testing error is also reported in the last three columns. The testing errors are measured for the testing geometries three, five, and nine, denoted as G3, G5, and G9, respectively. The accuracy of the given surrogate model is measured with respect to the numerical model.

We performed the reference experiment (‘E0’) for the simplest set of features where the effect of cooling from the surface is captured directly through the distances without any weighing function:

(14) $$\begin{array}{cc} \; & I_{s}=\sum_{i=1}^{N_{surf}}d_{i}\\ \; & I_{b}=d_{b} \end{array}$$

where $d_{i}$ are the distances of the *i*th points on the free surfaces from the point of interest and $N_{surf}$ is the number of points on the surface. We selected this feature set based on our previous work, where we tackle simpler geometries of increasing length [17]. The raw distances are also used in other SMs in AM [12]. Nevertheless, for our set of geometries, these features were insufficient for the model to converge, given the medium size of data. The neural network-based surrogate model did not converge within a reasonable range of the hyper-parameters (i.e., epochs < 300 and number of hidden nodes < 100). In this reference experiment, we used all data—including boundaries and unconsolidated points.

In the first experiment (‘E1’), we simplified the training data by excluding the part boundaries and unconsolidated points. These two types of data may be challenging as their behaviors are significantly different and it may be difficult to capture the pattern in the single SM. In ‘E1’, we additionally modified the features related to the distances from the cooling surfaces. We added the weighting function: $\left(\sum exp\left(-d^{2}\right)\right)$. With these two modifications, we report the ANN to converge. The resulting ANN consists of 50 nodes on a single hidden layer. However, the average testing error is relatively high, 15% across three geometries (G3, G5, and G9).

In experiment ‘E2’, we introduced the normalization factor 4α, which has a unit of m${}^{2}$/s and is inspired by the closed-form solution of the heat equation. As a result of this change, we observe an improvement in training and testing in ‘E2’ with a simpler ANN. In this case, only 25 hidden nodes are required to attain an average testing error of 7.5% across three tested geometries.

Encouraged by results from ‘E2’, in the next experiment—‘E3’—we increased the complexity of the data by including the boundary points. The resulting error (7.0%) did not significantly change, but the number of hidden nodes increased to 45.

To mitigate this effect, we tested two approaches. In the first approach, ‘E4’, we doubled the size of HIZ and increased it from 0.8 to 1.6 mm. This change was motivated by capturing more local information and the associated thermal signature. However, we did not notice any significant improvement in the testing error (5.8%) or the number of hidden nodes, which remained similar (40). This experiment increased our confidence that our features are capturing sufficient information about the thermal signature from the cooling surfaces. In the second approach, we introduced an additional feature that captures more temporal information (as opposed to more spatial information as in the first approach, ‘E4’). We include one more feature that computes the relative time of layer deposition. This configuration is marked as ‘E5’. In this case, we observe the number of hidden nodes reduced to 25 as well as reduced testing error (by 5.5%). Although the improvement is not significant, we can achieve better results with a smaller sized ANN compared to ‘E3’.

In the final iteration (‘E6’), we included the unconsolidated points through a classifier. Using the same set of features, we train the decision trees to determine if the point exhibits a lack of consolidation or not. With such setup, overall error slightly increased (7.3%). However, in this experiment, the model handles the complete set of points. In particular, the model is comprehensive to predict the consolidation degree for all points in the geometry. Altogether, we demonstrate that physics-inspired feature engineering not only reduces the number of required inputs for the model but also helps the learning process and improves model accuracy.

## 6. Conclusions and Future Work

In this paper, the features capturing the effect of the geometry and the printing pattern are presented and evaluated with a good performance of the corresponding SM. The performance of the SM was evaluated based on the series of geometries with high accuracy of 90%. Our good performance highlights the importance of feature engineering for robust surrogate model construction. The key observation from this study is that, when data are not readily available, one can build a reliable model by embedding more physics-based aspects in the set of features while avoiding a more complex model. Apart from high accuracy, our model offers four orders of magnitude faster prediction in comparison with the numerical methods. Such excellent performance opens avenues for real-time prediction. Finally, although this work focused on the FFF process, it can be transferred to other thermally driven processes in AM family.

## Figures and Tables

**Table 1:** Current state-of-the-art surrogate models in additive manufacturing.

Paper	AM Process	Features (Input of SM)	Output of SM	Reported Data Set	Model
Mozaffar [12]	DED	Laser power, scan speed, toolpath, geometry	Temperature (time series)	250,000	RNN
Stathatos [16]	LBAM	Trajectory descriptors	Temperature (time series)	54,450	Iterative ANN
Francis [13]	LBAM	Thermal images	Distortion field	21,818	CNN
Our work [17]	FFF	Distance from cooling surfaces	Temperature (time series)	12,000	ANN
Tapia [10]	L-PBF	Scan speed, laser power, beam size	Melt pool depth (scalar)	96	Gaussian process
Wang [9]	SLA	Geometry as grid connection	Pull up stress (scalar)	60	ANN

**Table 2:** List of the final features with the formulae provided and the link to reference figure depicting the corresponding feature.

Category	Associated Characteristic		Engineered Features	Ref.
Geometry	Heat sinks	Weighted effective distance	$I_{s}=\sum_{i=1}^{N_{surf}}exp\left(\frac{-d_{i}^{2}}{4\alpha }\right)$	Figure 2
	Heat source	Weighted effective distance	$I_{h}=\sum_{i=1}^{N_{surf}}exp\left(\frac{-d_{i}^{2}}{4\alpha }\right)$	Figure 2
		Effective area	$I_{l}=A_{input}/A_{HIZ}$	Figure 2
	Base	Weighted effective distance	$I_{b}=exp\left(\frac{-d_{b}^{2}}{4\alpha }\right)$	Figure 2
Printing pattern	Next road		$I_{n}=\frac{1}{|t_{d}-t_{n}|}$	Figure 3
	Previous road		$I_{p}=\frac{1}{|t_{d}-t_{p}|}$	Figure 3
	Layer		$I_{l}=\frac{1}{|t_{d}-t_{l}|}$	Figure 3

**Table 3:** Accuracy and speed of the surrogate model.

Geometry	Time (s)		% Accuracy
	Numerical Model	SM	
One (G1)	45,780	5.9	96.4
Two (G2)	122,210	5.8	95.5
Three (G3)	121,494	4.3	91.4
Four (G4)	74,701	6.0	95.0
Five (G5)	121,029	8.1	95.2
Six (G6)	171,000	7.3	93.6
Seven (G7)	49,646	2.4	89.0
Eight (G8)	239,931	4.4	93.1
Nine (G9)	172,732	3.9	91.9
Zero (G0)	186,496	4.0	92.4

**Note:** The surrogate model is on an average 25,000 times faster in predicting the bond strength than the numerical model.

**Table 4:** Engineering the features.

Runs	Features			Data Set		ANN Size	Testing Error		
	Distance Related Features	Size of HIZ (mm)	Extra Feature	Boundary	Unconsolidated Points		G3	G5	G9
E0	∑d	0.8	-	included	included	-	-	-	-
E1	$\sum exp(-d^{2})$	0.8	-	excluded	excluded	50	0.17	0.12	0.16
E2	$\sum \exp(-d^{2}/4\alpha )$	0.8	-	excluded	excluded	25	0.072	0.058	0.094
E3	$\sum exp(-d^{2}/4\alpha )$	0.8	-	**included**	excluded	45	0.068	0.056	0.087
E4	$\sum exp(-d^{2}/4\alpha )$	**1.6**	-	included	excluded	40	0.056	0.045	0.073
E5	$\sum exp(-d^{2}/4\alpha )$	1.6	$1/t_{\mathrm{layer}}$	included	excluded	25	0.058	0.042	0.065
E6	$\sum exp(-d^{2}/4\alpha )$	1.6	$1/t_{layer}$	included	**classifier**	25	0.088	0.048	0.082

**Note:***‘Runs’* correspond to the experiments conducted serially to improve the features. *‘ANN Size’* corresponds to the number of hidden nodes in the single hidden layer shallow neural network. *‘G#’* represents the testing geometries corresponding to the digits.

## Data Availability

The data presented in this study are available on reasonable request from the corresponding author.

## Appendix A. Dimensions of the Digits

All of the specimen consist of eight layers, each layer being 0.2 mm thick with dimensions given in Figure A1. The layers are built by depositing roads of 0.2 mm height and 0.4 mm thickness. All Gcodes are included in the data section.

## References

[1] Atif Yardimci M., Güçeri S. (1996). Conceptual framework for the thermal process modelling of fused deposition. Rapid Prototyp. J..

[2] Li L. (2002). Analysis and Fabrication of FDM Prototypes with Locally Controlled Properties.

[3] Costa S., Duarte F., Covas J. (2015). Thermal conditions affecting heat transfer in FDM/FFE: A contribution towards the numerical modelling of the process. Virtual Phys. Prototyp..

[4] Rodríguez J.F., Thomas J.P., Renaud J.E. (2001). Mechanical behavior of acrylonitrile butadiene styrene (ABS) fused deposition materials. Experimental investigation. Rapid Prototyp. J..

[5] Zhou X., Hsieh S.J., Ting C.C. (2018). Modelling and estimation of tensile behaviour of polylactic acid parts manufactured by fused deposition modelling using finite element analysis and knowledge-based library. Virtual Phys. Prototyp..

[6] Roy M., Yavari R., Zhou C., Wodo O., Rao P. (2019). Prediction and Experimental Validation of Part Thermal History in the Fused Filament Fabrication Additive Manufacturing Process. J. Manuf. Sci. Eng..

[7] Stockman T., Schneider J.A., Walker B., Carpenter J.S. (2019). A 3D Finite Difference Thermal Model Tailored for Additive Manufacturing. JOM.

[8] Neiva E., Badia S., Martín A.F., Chiumenti M. (2018). A scalable parallel finite element framework for growing geometries. application to metal additive manufacturing. arXiv.

[9] Wang J., Das S., Zhou C., Rai R. Data-Driven Simulation for Fast Prediction of Pull-Up Process in Bottom-Up Stereo-Lithography. Proceedings of the ASME 2016 International Design Engineering Technical Conferences and Computers and Information in Engineering Conference, American Society of Mechanical Engineers.

[10] Tapia G., Khairallah S., Matthews M., King W.E., Elwany A. (2018). Gaussian process-based surrogate modeling framework for process planning in laser powder-bed fusion additive manufacturing of 316L stainless steel. Int. J. Adv. Manuf. Technol..

[11] Yan W., Lin S., Kafka O.L., Lian Y., Yu C., Liu Z., Yan J., Wolff S., Wu H., Ndip-Agbor E. (2018). Data-driven multi-scale multi-physics models to derive process–structure–property relationships for additive manufacturing. Comput. Mech..

[12] Mozaffar M., Paul A., Al-Bahrani R., Wolff S., Choudhary A., Agrawal A., Ehmann K., Cao J. (2018). Data-driven prediction of the high-dimensional thermal history in directed energy deposition processes via recurrent neural networks. Manuf. Lett..

[13] Francis J., Bian L. (2019). Deep Learning for Distortion Prediction in Laser-Based Additive Manufacturing using Big Data. Manuf. Lett..

[14] Jiang J., Hu G., Li X., Xu X., Zheng P., Stringer J. (2019). Analysis and prediction of printable bridge length in fused deposition modelling based on back propagation neural network. Virtual Phys. Prototyp..

[15] Viana F.A., Gogu C., Haftka R.T. Making the most out of surrogate models: Tricks of the trade. Proceedings of the ASME 2010 International Design Engineering Technical Conferences and Computers and Information in Engineering Conference, American Society of Mechanical Engineers Digital Collection.

[16] Stathatos E., Vosniakos G.C. (2019). Real-time simulation for long paths in laser-based additive manufacturing: A machine learning approach. Int. J. Adv. Manuf. Technol..

[17] Roy M., Wodo O. (2020). Data-driven modeling of thermal history in additive manufacturing. Addit. Manuf..

[18] Roy M., Wodo O. (2019). Quality assurance in additive manufacturing of thermoplastic parts: Predicting consolidation degree based on thermal profile. Int. J. Rapid Manuf..

[19] Carslaw H.S., Jaeger J.C. (1959). Conduction of Heat in Solids.

[20] Tsao C.W., DeVoe D.L. (2009). Bonding of thermoplastic polymer microfluidics. Microfluid. Nanofluidics.

[21] Khosravani M.R., Reinicke T. (2020). Effects of raster layup and printing speed on strength of 3D-printed structural components. Procedia Struct. Integr..

[22] Nurizada A., Kirane K. (2020). Induced anisotropy in the fracturing behavior of 3D printed parts analyzed by the size effect method. Eng. Fract. Mech..

[23] Bastien L., Gillespie J. (1991). A non-isothermal healing model for strength and toughness of fusion bonded joints of amorphous thermoplastics. Polym. Eng. Sci..

[24] Williams M.L., Landel R.F., Ferry J.D. (1955). The temperature dependence of relaxation mechanisms in amorphous polymers and other glass-forming liquids. J. Am. Chem. Soc..

[25] Bartolai J., Simpson T.W., Xie R. (2018). Predicting strength of additively manufactured thermoplastic polymer parts produced using material extrusion. Rapid Prototyp. J..

[26] Liu H., Motoda H. (2007). Computational Methods of Feature Selection.

[27] Koller D., Sahami M. (1996). Toward Optimal Feature Selection.

[28] Bahnsen A.C., Aouada D., Stojanovic A., Ottersten B. (2016). Feature engineering strategies for credit card fraud detection. Expert Syst. Appl..

[29] Seide F., Li G., Chen X., Yu D. Feature engineering in context-dependent deep neural networks for conversational speech transcription. Proceedings of the 2011 IEEE Workshop on Automatic Speech Recognition & Understanding.

[30] Garla V.N., Brandt C. (2012). Ontology-guided feature engineering for clinical text classification. J. Biomed. Inform..

[31] Dan Foresee F., Hagan M.T. Gauss-Newton approximation to Bayesian learning. Proceedings of the International Conference on Neural Networks (ICNN’97).

